# The origins of water conducting tissues as clues to bryophyte evolution

**DOI:** 10.1093/plphys/kiag604

**Published:** 2026-08-13

**Authors:** Alison R Gill

**Affiliations:** Assistant Features Editor, Plant Physiology, American Society of Plant Biologists; Australian Research Council Centre of Excellence in Plants for Space, Adelaide University, Urrbrae, SA 5064, Australia

During the early evolution of land plants, the major lineages that gave rise to modern tracheophytes (vascular plants with specialized water-conducting tissues, true roots, stems, and leaves; Fig. 1a) and bryophytes (mosses, liverworts, and hornworts, without true vascular tissues; Fig. 1a) diverged from a common ancestor. The genes and genetic changes underlying bryophyte evolution and adaptation to life on land have been debated, partly due to uncertainty about the phylogenetic relationships among bryophytes and their relationships to tracheophytes (Dong et al. 2025). Recent genomic and transcriptomic evidence has resolved bryophytes as a monophyletic lineage sister to all living tracheophytes (Harris et al. 2020; Shen et al. 2025; Xue et al. 2025). Central to the uncertainty was whether shared traits in both bryophytes and vascular plants, such as water-conducting tissues, evolved independently through convergent evolution or whether bryophytes acquired their simpler characteristics through reductive evolution from a more complex common ancestor of land plants (Fig. 1b).

**Figure 1:**
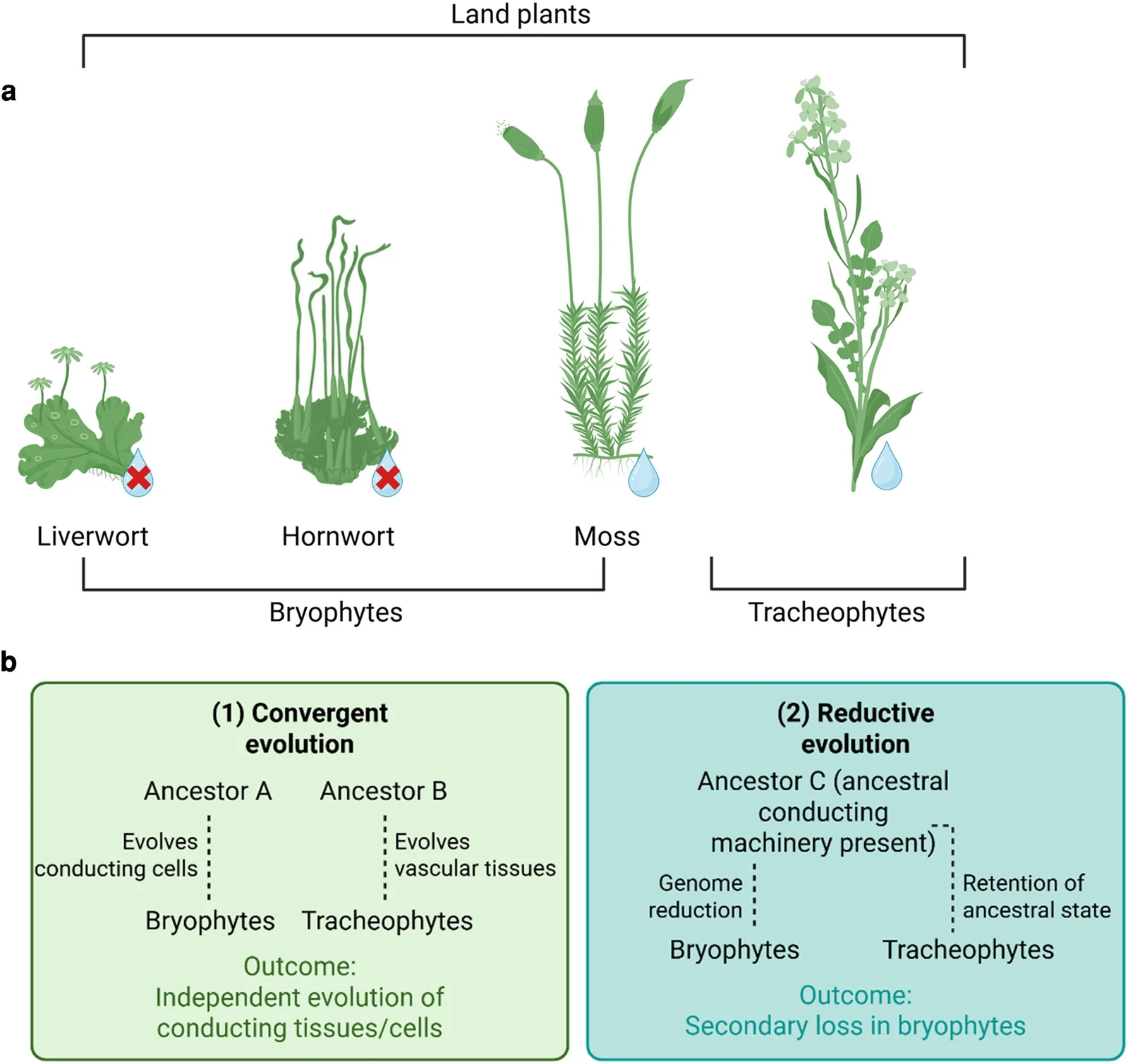
(a) Major land plant lineages discussed in Bowles (2026). Bryophytes comprise liverworts, hornworts, and mosses, whereas tracheophytes are represented here by a flowering plant (but also comprise lycophytes, ferns, and gymnosperms). The blue water droplet indicates the presence of specialized water-conducting cells in mosses and true xylem in tracheophytes, while the crossed droplet indicates the absence of specialized water-conducting tissues. (b) Hypotheses for the evolution of water-conducting tissues. (1) Convergent evolution: water-conducting structures evolved independently in bryophytes and tracheophytes. (2) Reductive evolution: an ancestor possessed conducting machinery and bryophytes subsequently lost components through genome reduction and simplification. Created in BioRender, hornwort figure adapted from The Chinese University of Hong Kong (no date).

The vascular system is one of the most vital and transformative plant innovations, underpinning the successful establishment and diversification of plant life on land five hundred million years ago (Dong et al. 2025). Two mechanisms of water transport have evolved since this transition: the vascular tissues of vascular plants and the water-conducting cells of some mosses (Brodribb et al. 2020). The question remains as to whether water-conducting tissues evolved only once or on multiple occasions, with implications for bryophyte phylogeny.

In a recent paper published in *Plant Physiology*, Bowles (2026) investigated genome evolution within bryophyte lineages to understand the origins of water-conducting tissues. Bowles used phylogenomic approaches to generate a species tree based on ortholog inference and identified novel, duplicated and lost gene groups (orthogroups) based on molecular data from 160 species. Bowles then investigated genes associated with xylem development and performed ancestral-state reconstruction to infer the evolutionary history of these traits.

Bowles discovered that the water-conducting system did not emerge as a single complex innovation, but rather through small, incremental genetic changes that began before today's major land plant diverged from their common ancestor and evolved into separate groups. The ancestral plants already had some of the genetic tools for water transport before vascular plants appeared. Over time, these genes were modified and improved through a continuum of functional diversification, eventually leading to the efficient vascular systems seen in modern vascular plants.

Bowles found many convergent gene duplications between mosses and vascular plants that were involved in a range of processes, including metabolism, secondary metabolite biosynthesis, signal transduction, response to environmental stressors, growth, and development. Vascular NAC domain transcription factors, which are types of regulatory genes, have functions in the development of both water-conducting cells and xylem (Endo et al. 2015), and were conserved across bryophytes and tracheophytes. This observation is consistent with Xu et al. (2014) who found that NAC transcriptional regulation was conserved across moss and *Arabidopsis thaliana*, suggesting a key role in the adaptation of plants to land.

Hornworts, unlike mosses, do not have water-conducting tissues and lack much of the conserved genetic network underlying xylem differentiation and water-conducting cell specialization. For example, vascular NAC domain transcription factors are absent in hornworts. Bowles established that hornworts had lost SND2-5 (SECONDARY NAC DOMAIN Transcription Factors) involved in xylem fiber differentiation; SND2-4, which controls vascular tissue patterning and secondary cell wall formation; and SHOOT MERISTEMLESS (STM), which maintains meristematic identity in the shoot and provides the developmental context from which vascular tissues are specified. Other lost gene families were involved in cellular development, sieve element development, and photosynthetic function. Hornworts were found to be the most genomically reduced bryophyte lineage, with 1,696 gene families absent, and to exhibit the greatest rates of convergent gene duplication with vascular plants. Extensive developmental genes were lost, coinciding with the absence of water-conducting cells.

The findings of Bowles (2026) are consistent with the hypotheses that hornworts have simplified genomes, that there is convergent gene duplication in vascular plants and water-conducting mosses, and that there are specific innovations of vascular plants. The results also suggest that bryophytes did not arise directly from a simple, primitive plant and remain as unchanged remnants of this early land plant. Instead, they evolved from a more complex ancestor and have continued to evolve, with extensive gene loss and simplification. The phylogenomic analyses reported by Bowles suggest that these earliest land plants lacked water-conducting tissues and, instead, that these tissues evolved later in multiple plant lineages.

In summary, water-conducting tissues did not appear all at once as a completely new innovation. Instead, they evolved gradually through repeated adaptations of genetic tools already present in plants. Incremental molecular changes over time led to this major morphological innovation in both bryophytes and tracheophytes, enabling the establishment and diversification of plants on land.

## Recent related articles in *Plant Physiology*

- Romani et al. (2026) investigated an ancestral developmental role for Class I homeodomain-leucine zipper (*C1HDZ)* genes. As part of the Focus Collection on Early Land Plant Evolution, this paper explored the functions of C1HDZ in the liverwort *Marchantia polymorpha* as its role in bryophytes is poorly understood.
- Li et al. (2024) explored the evolutionary history and functional divergence of aluminum-activated malate transporters and slow anion channels, and their important role in the adaptation and diversification of vascular plants.

## Data Availability

No new data were generated or analyzed for this News and Views article.
